# Clinical outcomes of thyroid function in patients with thyrotropin receptor blocking antibody-positive hypothyroidism

**DOI:** 10.1210/jendso/bvag147

**Published:** 2026-06-30

**Authors:** Rei Hirose, Jaeduk Yoshimura Noh, Natsuko Watanabe, Tatsuya Iida, Toshino Suzuki, Masahiro Ichikawa, Masakazu Koshibu, Nami Suzuki, Masako Matsumoto, Miho Fukushita, Ai Yoshihara, Kiminori Sugino, Koichi Ito

**Affiliations:** Department of Internal Medicine, Ito Hospital, Tokyo 150-8308, Japan; Department of Internal Medicine, Ito Hospital, Tokyo 150-8308, Japan; Department of Internal Medicine, Ito Hospital, Tokyo 150-8308, Japan; Department of Internal Medicine, Ito Hospital, Tokyo 150-8308, Japan; Department of Internal Medicine, Ito Hospital, Tokyo 150-8308, Japan; Department of Internal Medicine, Ito Hospital, Tokyo 150-8308, Japan; Department of Internal Medicine, Ito Hospital, Tokyo 150-8308, Japan; Department of Internal Medicine, Ito Hospital, Tokyo 150-8308, Japan; Department of Internal Medicine, Ito Hospital, Tokyo 150-8308, Japan; Department of Internal Medicine, Ito Hospital, Tokyo 150-8308, Japan; Department of Internal Medicine, Ito Hospital, Tokyo 150-8308, Japan; Department of Surgery, Ito Hospital, Tokyo 150-8308, Japan; Department of Surgery, Ito Hospital, Tokyo 150-8308, Japan

**Keywords:** thyrotropin receptor blocking antibody, hypothyroidism, Graves’ disease, thyroid volume

## Abstract

**Context:**

Thyrotropin receptor blocking antibodies (TBAbs) are uncommon but not a rare cause of hypothyroidism. However, clinical outcomes of thyroid function in patients with TBAb-positive hypothyroidism remain unclear.

**Objective:**

The aim of this study was to explore the clinical outcomes of thyroid function in patients with TBAb-positive hypothyroidism and investigate factors associated with hypothyroidism outcomes.

**Methods:**

A single-center retrospective observational study was conducted from 2024 to 2025. Overall, 137 patients with TBAb-positive hypothyroidism without Graves’ disease (Non-GD group) and 181 with TBAb-positive hypothyroidism during or after antithyroid drug therapy for Graves’ disease (Prior GD group), almost all Japanese, were included. Thyroid functional outcomes were assessed in all patients; serial TBAb measurements were available for 31% and 36% of the patients, respectively. The main outcome was thyroid function at the final visit after a median follow-up period of 6.8 years. Clinical factors associated with hypothyroidism outcomes were analyzed using logistic regression analysis.

**Results:**

Hypothyroidism persisted in 84% and 43% of the patients in the Non-GD and Prior GD groups, respectively. In both groups, persistent TBAb positivity was associated with hypothyroid outcomes (Non-GD: univariate analysis, odds ratio [OR], 3.51; 95% CI, 1.14-40.6; *P* = .013; Prior GD: univariate analysis, OR, 6.75; 95% CI, 1.87-24.4; *P* = .0016; multivariate analysis, OR, 6.88; 95% CI, 1.89-25.1; *P* = .0015).

**Conclusion:**

Most patients with TBAb-positive hypothyroidism remain hypothyroid over the long term, whereas outcomes are more heterogeneous in patients with prior Graves’ disease who develop TBAb-positive hypothyroidism. Persistent TBAb positivity is associated with the hypothyroidism outcomes.

Thyrotropin (TSH) receptor blocking antibody (TBAb; also referred to as TSH receptor–stimulating blocking antibody) is a type of TSH receptor (thyroid-stimulating hormone receptor [TSHR]) antibody that binds to the TSH receptor and blocks the action of TSH. TBAb is one of the causes of hypothyroidism; however, hypothyroidism does not always persist in some patients with positive TBAb. Furthermore, TBAb can coexist with TSH receptor–stimulating antibody (TSAb), and their interplay may cause complex clinical conditions in thyroid dysfunction, such as transitions between hyperthyroidism and hypothyroidism or swings between the 2 states [1-5].

TBAb is not rare in patients with thyroid dysfunction. It has been detected in approximately 10% to 15% of patients with autoimmune thyroid disease, 10% to 13% of those with hypothyroidism, 17.8% of those with severe hypothyroidism (low thyroxine [T4] and TSH >20 mU/L), 8% to 13% of those with Hashimoto's thyroiditis, 4% to 8% of those with Graves’ disease (GD), and up to 38.8% of those with childhood-onset atrophic autoimmune thyroiditis [6-14]. Moreover, 5% to 20% of patients with GD treated with antithyroid drugs develop hypothyroidism, and approximately one-third of these patients are attributable to TBAb [15-17]. However, the longitudinal clinical course of thyroid function in patients with hypothyroidism or GD who are TBAb-positive has scarcely been reported. To date, only 2 studies by Takasu et al have investigated the outcomes of TBAb-positive hypothyroidism in adults, both of which were based on small patient cohorts [10, 18]. In these reports, more than half of the patients exhibited persistent hypothyroidism, whereas the remainder reached euthyroidism, and a few developed GD. Persistent TBAb positivity and an atrophic thyroid have been suggested to be associated with the hypothyroidism outcomes. Among pediatric patients, 1 patient with atrophic autoimmune thyroiditis has been reported in whom TBAb disappeared during follow-up and subsequently thyroid function recovered from hypothyroidism to euthyroidism [19]. No previous studies have reported the outcomes of TBAb-positive individuals who develop hypothyroidism in patients with GD. Accordingly, in the present study, we aimed to investigate the outcomes of thyroid function in a relatively large cohort of patients with TBAb-positive hypothyroidism with or without GD, analyze the factors associated with the clinical outcomes, and provide insights that may aid in the management of TBAb-positive patients.

## Methods

### Participants

**Figure 1:**
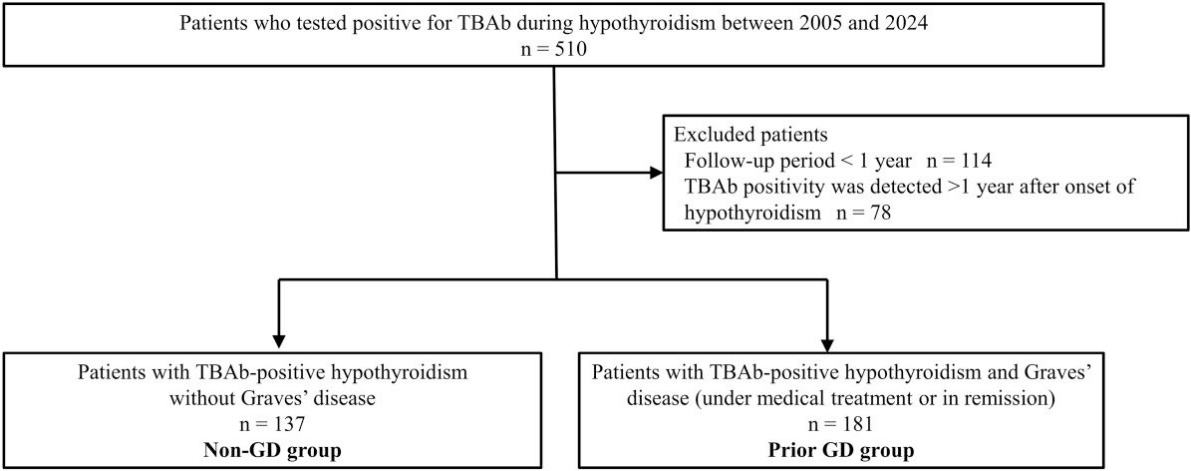
Flowchart of participant selection. TBAb, thyrotropin receptor blocking antibody.

This study included 510 patients with positive TBAb during hypothyroidism at our hospital between 2005 and 2024, none of whom had a history of thyroid surgery or radioactive iodine therapy. Patients with a follow-up period of <1 year after TBAb positivity and those whose TBAb positivity was detected >1 year after the diagnosis of hypothyroidism were excluded. The remaining 318 patients were divided into 2 groups according to the presence or absence of a history of GD: 137 patients with TBAb-positive hypothyroidism without a history of GD (Non-GD group) and 181 patients with TBAb-positive hypothyroidism and a history of GD, either under medical treatment or in remission (Prior GD group) (Fig. 1).

This retrospective study was approved by the Ethics Committee of Ito Hospital (approval number: 459) and was conducted in accordance with the Declaration of Helsinki and current legal laws in Japan. An opt-out approach was used to provide patients with the opportunity to refuse participation in this study.

### Outcomes of thyroid function

Thyroid functional outcomes were classified according to serum TSH and free T4 (FT4) levels. Hyperthyroidism was defined as a suppressed TSH level with an elevated or normal FT4 level, euthyroidism as normal TSH and FT4 levels, and hypothyroidism as an elevated TSH level with a decreased or normal FT4 level. Patients receiving antithyroid drug therapy who had normal TSH and FT4 levels were classified as hyperthyroid, whereas patients receiving levothyroxine replacement therapy who had normal TSH and FT4 levels were classified as hypothyroidism.

**Figure 2:**
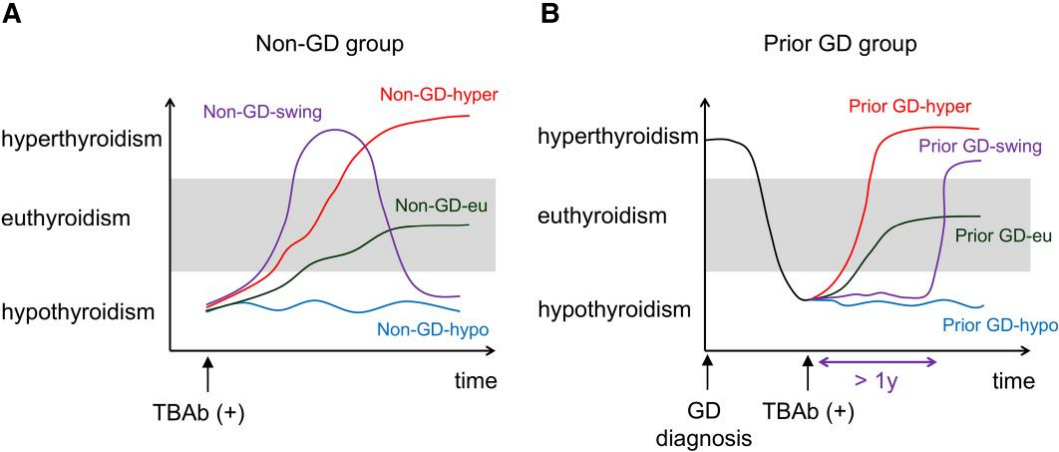
Clinical course of each subgroup according to the outcomes of thyroid function in the Non-GD (A) and Prior GD (B) groups. The Non-GD-swing group comprised patients who developed thyrotoxicosis after the initial diagnosis of hypothyroidism, followed by a subsequent return to hypothyroidism. The Prior GD-swing group comprised patients who developed hypothyroidism persisting for >1 year after the initial diagnosis of GD, followed by a subsequent return to hyperthyroidism. The clinical course of the Prior GD-BR group is not shown. TBAb, thyrotropin receptor blocking antibody; GD, Graves’ disease.

The Non-GD group was further classified into 4 categories according to the thyroid functional outcomes at the final follow-up visit: hyperthyroidism (Non-GD-hyper), euthyroidism (Non-GD-eu), hypothyroidism (Non-GD-hypo), and hyperthyroidism followed by hypothyroidism (Non-GD-swing) (Fig. 2A). Similarly, the Prior GD group was further classified into 5 categories: hyperthyroidism (Prior GD-hyper), euthyroidism (Prior GD-eu), hypothyroidism (Prior GD-hypo), hypothyroidism for >1 year followed by hyperthyroidism (Prior GD-swing), and block-and-replacement therapy (Prior GD-BR) (Fig. 2B). For multivariate analysis, the Prior GD-eu, Prior GD-hyper, and Prior GD-swing groups were merged into a single group (Prior GD-non-hypo) based on the shared features of nonpersistent hypothyroidism.

### Measurement of laboratory data

Performance characteristics of the assays used for TSHR-directed autoantibody measurements are shown in Table S1 [20]. Until June 2014, the levels of TSAb and TBAb were determined using a cell-based bioassay kit in combination with radioimmunoassay (RIA) (Yamasa Corp., Chosi, Chiba, Japan) [21, 22]. The reference ranges were ≤180% for TSAb (RIA) and ≤45.6% for TBAb (RIA). Between July 2014 and February 2023, a newly developed bioassay kit coupled with an enzyme immunoassay (EIA) (Yamasa Corp.) was used to measure TSAb and TBAb levels [22, 23]. The normal limits of the TSAb (EIA) and TBAb (EIA) were ≤120% and ≤31.7%, respectively. As extremely high TSAb values can yield false-positive TBAb results, measurements of TBAb (RIA) and TBAb (EIA) were considered unreliable when TSAb (RIA) was >600% or TSAb (EIA) was >1500% [21, 23]. Therefore, TBAb (RIA) positivity was assessed only when TSAb (RIA) was ≤600% and TBAb (EIA) positivity only when TSAb (EIA) was ≤1500%. Since March 2023, TBAb levels have been measured using a novel bioassay kit that incorporates a cyclic adenosine monophosphate biosensor (Bio; Yamasa Corp.) [24]. This system quantifies the stimulatory activity toward TSHR as a stimulating index (SI) and evaluates the inhibitory activity as a blocking index (BI), independent of stimulation. Consequently, this assay eliminates the confounding effects of stimulatory activity when assessing TBAb. TBAb positivity was defined as TBAb (Bio) ≥13.1% together with BI ≥8.0. Since March 2023, TSAb levels have been measured using a novel biosensor-based assay kit (Bio; Yamasa Corp.) [25, 26]. The normal limit of the TSAb (Bio) was <110%.

The TSH receptor antibody (TRAb) was measured by the Cosmic TRAb CT (Cosmic Ltd., Tokyo, Japan) using RIA until September 2008 [27], after which it was measured using electrochemiluminescence immunoassay (ECLIA) (ECLusys TRAb; Roche Diagnostics, Basel, Switzerland) [28]. The reference ranges at our facility were set as follows: TRAb (RIA) ≤10% and TRAb (ECLIA) <2.0 IU/L.

The FT4 and TSH levels were measured using ECLIA kits (ECLusys FT4 and TSH; Roche Diagnostics). The reference ranges at our facility were set as follows: FT4 0.8-1.6 ng/dL and TSH 0.2-4.5 μIU/mL. The FT4 values were converted to SI units (pmol/L) using the following conversion factor: 1 ng/dL = 12.87 pmol/L. Thyroglobulin antibody (TgAb) and thyroid peroxidase antibody (TPOAb) levels were determined using RIA until May 2006, after which they were measured using ECLIA. The reference ranges at our facility were set as follows: TgAb (RIA) <0.3 U/mL, TPOAb (RIA) <0.3 U/mL, TgAb (ECLIA) ≤40 IU/mL, and TPOAb (ECLIA) ≤28 IU/mL.

### Measurement of thyroid volume

Thyroid volume (TV) was determined using ultrasonography. First, the length (l), width (w), and depth (d) of each thyroid lobe were measured (in centimeters). Subsequently, TV was calculated using the following formula: TV (mL) = (0.7365 × right lobe l × w × d + 0.7412 × left lobe l × w × d) − 0.55, as previously reported [29].

### Statistical analyses

Data are presented as medians and ranges for continuous variables and as numbers or percentages for categorical variables. Differences in clinical parameters between the 2 groups were analyzed using the Wilcoxon rank-sum test for continuous variables and Pearson's chi-squared or Fisher's exact test for categorical variables. Differences in clinical parameters among the 3 groups were analyzed using the Kruskal–Wallis test, followed by Dunn's test for continuous variables or Pearson's chi-squared test for categorical variables. The Wilcoxon signed-rank test was used to compare the paired parameters. A logistic regression model was used for risk analysis, with Firth's penalized likelihood method applied in cases of complete separation. In the logistic regression analyses, all variables except for sex and transition of TBAb status were treated as continuous variables. To improve interpretability, selected variables were rescaled so that odds ratios (ORs) represent clinically meaningful increments (eg, per 10 or 100 units), depending on the distribution of each variable. In multivariate analyses, patients with missing data for any covariates were excluded, and a complete-case approach was used. Owing to the limited sample size, a multivariate model was constructed with only 2 explanatory variables to minimize overfitting. The longitudinal change in TBAb levels was selected as the primary explanatory variable of interest, and the observation period was included as the most important confounding factor. A *P*-value <.05 was considered statistically significant. All analyses were performed using the JMP software version 12.0.1 (SAS Institute Inc., Cary, NC, USA).

## Results

### Outcomes of thyroid function

**Figure 3:**
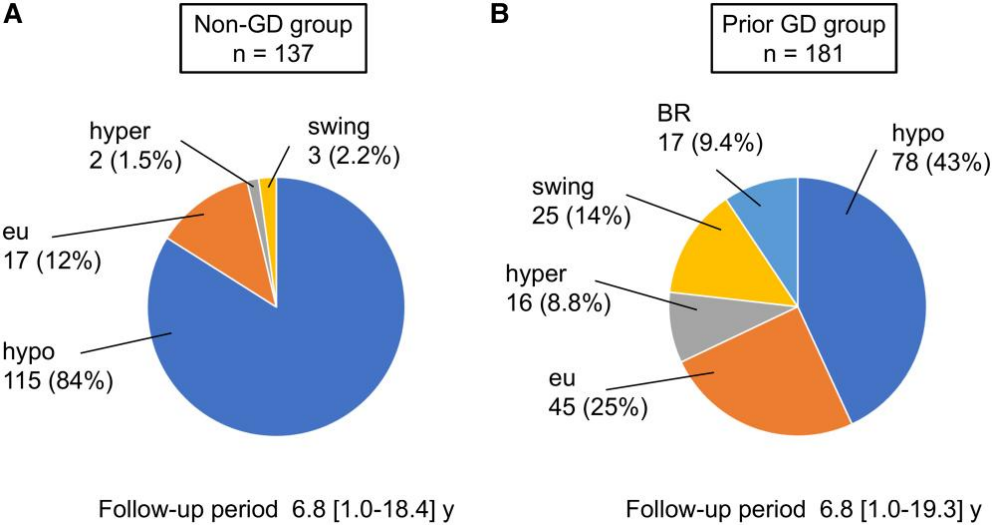
Outcomes of thyroid function in the Non-GD (A) and Prior GD (B) groups.

In the Non-GD group, hypothyroidism persisted in most patients (84%) (Fig. 3A). A total of 12% of the patients achieved euthyroidism, whereas hyperthyroidism or a swinging course was rare. Notably, all patients with these outcomes (hyperthyroidism *n* = 2; swinging course *n* = 3) were newly diagnosed with GD during the follow-up period.

In the Prior GD group, clinical outcomes were more heterogeneous; persistent hypothyroidism was observed in 43% of the patients (Fig. 3B). Patients with outcomes of hyperthyroidism or a swinging course were more frequent than in patients with TBAb-positive hypothyroidism without GD. Treatment with the block-and-replacement regimen was administered to 9.4% of the patients. The median follow-up duration was 6.8 years in both groups.

### Clinical characteristics of patients

In the Non-GD group, there were no significant differences in TBAb, TSAb, TRAb, thyroid function, or TV at the time of hypothyroidism diagnosis between the Non-GD-hypo and Non-GD-eu groups (Table 1). All patients in the Non-GD-hyper and Non-GD-swing groups were positive for TSAb, and TSAb-positive patients were also included in the Non-GD-hypo and Non-GD-eu groups. The TgAb (ECLIA) and TPOAb (ECLIA) levels tended to be higher in the Non-GD-hypo group than in the Non-GD-eu group, although the differences were not statistically significant.

**Table 1:** Characteristics of the non-GD group

	Non-GD-hypo (*n* = 115)	Non-GD-eu (*n* = 17)	Non-GD-hyper (*n* = 2)	Non-GD-swing (*n* = 3)	*P*-value hypo vs eu
Age (years)	50 [5-84]	51 [19-78]	36, 37	48, 65, 70	.75
Sex (F/M), *n* (%)	94 (82)/21 (18)	17 (100)/0 (0)	2 (100)/0 (0)	3 (100)/0 (0)	.073
Follow-up period after TBAb measurement (years)	6.5 [1.0-16.8]	7.9 [1.7-18.4]	1.8, 2.0	5.8, 9.2, 9.3	.12
FT4 (pmol/L)*^a^*	5.79 [0.51-18.1]	7.98 [1.03-15.8]	9.27, 15.4	6.82, 13.8, 14.2	.24
TSH (µIU/mL)*^a^*	77.5 [4.82-624]	83.3 [4.72-161]	14.0, 17.9	5.47, 24.5, 56.1	.27
Duration of hypothyroidism (years)	6.6 [1.1-17.0]	1.2 [0.01-9.6]	0.4, 0.9	4.0, 4.4, 9.3	<.0001
Thyroid autoantibodies					
TBAb (RIA) (%)	64 [47-100] (*n* = 57)	58 [47-99] (*n* = 11)	NA	NA	.43
TBAb (EIA) (%)	97.8 [33.3-105] (*n* = 52)	96.7 [50-102] (*n* = 6)	94, 95	89, 90, 98	.32
TBAb (Bio) (%)	74.3 [15.8-100] (*n* = 6)	NA	NA	NA	
SI	2.5 [0.8-7.5] (*n* = 6)	NA	NA	NA	
BI	17.2 [10.3-44.6] (*n* = 6)	NA	NA	NA	
TSAb (RIA) (%)*^a^*	140 [86-368] (*n* = 57)	140 [105-360] (*n* = 11)	NA	NA	.98
TSAb (EIA) (%)*^a^*	132 [91-1487] (*n* = 52)	140 [125-271] (*n* = 6)	138, 362	123.356, 1124	.39
TSAb (Bio) (%)*^a^*	1010 [107-7350] (*n* = 5)	NA	NA	NA	
TRAb (RIA) (%)*^a^*	56.7 [−0.3-97.0] (*n* = 20)	70.6 [32.7-87.4] (*n* = 4)	NA	NA	.79
TRAb (ECLIA) (IU/L)*^a^*	22.6 [0.3-40] (*n* = 89)	20.4 [0.4-40] (*n* = 11)	10.6, 37.8	15.8, 35.4, 40	.80
TgAb (RIA) (U/mL)*^b^*	2.9 [0.3-69] (*n* = 7)	6.1, 8.8 (*n* = 2)	NA	NA	.66
TgAb (ECLIA) (IU/mL)*^b^*	330.2 [10-4000] (*n* = 104)	51.8 [10-3979] (*n* = 15)	88.2 (*n* = 1)	28.9, 613, 2957	.066
TPOAb (RIA) (U/mL)*^b^*	12 [0.3-50] (*n* = 7)	8.1, 28 (*n* = 2)	NA	NA	1.0
TPOAb (ECLIA) (IU/mL)*^b^*	271.0 [5.7-600] (*n* = 104)	79.9 [5-600] (*n* = 15)	600 (*n* = 1)	18, 39, 226	.15
TV at the time of hypothyroidism diagnosis (mL)	12.8 [4.4-73.8] (*n* = 45)	3.9, 13.2, 14.3 (*n* = 3)	13.0, 24.0	3.6 (*n* = 1)	.58

Abbreviations: TBAb, thyrotropin receptor blocking antibody; FT4, free thyroxine; TSH, thyrotropin; RIA, radioimmunoassay; EIA, enzyme immunoassay; Bio, biosensor; SI, stimulating index; BI, blocking index; TSAb, thyrotropin receptor–stimulating antibody; TRAb, thyrotropin receptor antibody; ECLIA, electrochemiluminescence immunoassay; TgAb, thyroglobulin antibody; TPOAb, thyroid peroxidase antibody; TV, thyroid volume; NA, not available

^*a*^Measured at the time of TBAb measurement.

^*b*^Measured at the first visit.

In the Prior GD group, TBAb (RIA) levels were significantly higher in the Prior GD-hypo group than in the Prior GD-hyper group, whereas no significant differences were observed in TBAb (EIA) levels (Table 2). TSAb levels and thyroid function did not differ among the groups. TgAb (ECLIA) levels were significantly higher in the Prior GD-hypo and Prior GD-hyper groups than in the Prior GD-eu group. Similarly, TPOAb (ECLIA) levels tended to be higher, although the differences were not statistically significant. At the time of hypothyroidism diagnosis, TV was smaller in the Prior GD-hypo group than in the Prior GD-eu and Prior GD-hyper groups; however, the differences were not significant.

**Table 2:** Characteristics of the prior GD group

	(I) Prior GD-hypo (*n* = 78)	(II) Prior GD-eu (*n* = 45)	(III) Prior GD-hyper (*n* = 16)	Prior GD-swing (*n* = 25)	*P*-value		*P*-value	
					1 vs II vs III	I vs II	I vs III	II vs III
Age (years)	45 [19-83]	49 [19-86]	35 [10-67]	42 [18-63]	.029	1.0	.049	0.029
Sex (F/M), *n* (%)	69 (88)/9 (12)	36 (80)/9 (20)	12 (75)/4 (25)	22 (88)/3 (12)	.26			
Follow-up period after TBAb measurement (years)	7.1 [1.0-18.8]	6.4 [1.3-17.0]	5.2[1.1-19.3]	10.0 [2.1-18.7]	.66			
FT4 (pmol/L)*^a^*	8.62 [0.51-20.3]	7.85 [1.29-18.5]	7.98 [1.93-14.8]	8.88 [0.90-15.4]	.70			
TSH (µIU/mL)*^a^*	40.0 [4.97-264]	27.4 [4.7-111]	24.0 [5.53-186]	24.2 [4.56-121]	.31			
Thyroid autoantibodies								
TBAb (RIA) (%)	67 [46-100] (*n* = 38)	56 [47-97] (*n* = 36)	52 [47-64] (*n* = 7)	60 [47-95] (*n* = 15)	.0098	.055	.04	.77
TBAb (EIA) (%)	98 [43-108] (*n* = 41)	98 [67-106] (*n* = 9)	97 [54-101] (*n* = 7)	94 [72-101] (*n* = 10)	.72			
TBAb (Bio) (%)	NA	NA	41.2, 52.5 (*n* = 2)	NA				
SI	NA	NA	2.9, 5.1 (*n* = 2)	NA				
BI	NA	NA	8.1, 11.9 (*n* = 2)	NA				
TSAb (RIA) (%)*^a^*	182 [72-513] (*n* = 37)	207 [102-542] (*n* = 36)	309 [112-527] (*n* = 7)	247 [102-548] (*n* = 15)	.18			
TSAb (EIA) (%)*^a^*	159 [91-1089] (*n* = 39)	267 [108-1296] (*n* = 9)	220 [176-1275] (*n* = 7)	209 [99-1453] (*n* = 10)	.13			
TSAb (Bio) (%)*^a^*	NA	NA	2440, 5260 (*n* = 2)	NA				
TRAb (RIA) (%)*^a^*	49.9 [12.9-95.8] (*n* = 8)	46.6 [11.9-82.6] (*n* = 10)	44.4, 90.7 (*n* = 2)	65.1 [20.2-88.3] (*n* = 6)	.50			
TRAb (ECLIA) (IU/L)*^a^*	30.8 [0.6-40] (*n* = 61)	8.7 [0.8-40] (*n* = 29)	28.6 [1.7-40] (*n* = 13)	18.6 [1.5-40] (*n* = 16)	.023	.018	1	.65
TgAb (RIA) (U/mL)*^b^*	5.9 [0.3-100] (*n* = 10)	4.1 [0.3-12.6] (*n* = 6)	0.3, 8.6 (*n* = 2)	4 [0.3-100] (*n* = 4)	.67			
TgAb (ECLIA) (IU/mL)*^b^*	471 [12.2-4000] (*n* = 63)	31 [10-1263] (*n* = 39)	396 [10-4000] (*n* = 14)	247 [10-693] (*n* = 20)	<.0001	<.0001	1	.032
TPOAb (RIA) (U/mL)*^b^*	50 [1.2-50] (*n* = 10)	45.6 [0.3-50] (*n* = 6)	0.3, 50 (*n* = 2)	0.5 [0.3-30.9] (*n* = 4)	.66			
TPOAb (ECLIA) (IU/mL)*^b^*	308 [5.2-600] (*n* = 63)	53 [5-600] (*n* = 39)	262 [8.7-600] (*n* = 14)	196 [5.2-600] (*n* = 20)	.16			
TV upon hyperthyroidism (mL)	23.4 [10.5-52.0] (*n* = 15)	40.6 [16.9-49.5] (*n* = 3)	34.4 [18.9-43.4] (*n* = 5)	32.6, 126.6 (*n* = 2)	.43			

Abbreviations: TBAb, thyrotropin receptor blocking antibody; FT4, free thyroxine; TSH, thyrotropin; RIA, radioimmunoassay; EIA, enzyme immunoassay; Bio, biosensor; SI, stimulating index; BI, blocking index; TSAb, thyrotropin receptor–stimulating antibody; TRAb, thyrotropin receptor antibody; ECLIA, electrochemiluminescence immunoassay; TgAb, thyroglobulin antibody; TPOAb, thyroid peroxidase antibody; TV, thyroid volume; NA, not available

^*a*^Measured at the time of TBAb measurement.

^*b*^Measured at the first visit.

### Transition of TSHR-directed autoantibodies status and thyroid functional outcomes

In the Non-GD-hypo group, longitudinal changes in TBAb levels were observed in 35 patients, of whom 17 (49%) showed persistent TBAb positivity and 18 (51%) became negative (Table 3). In the Non-GD-eu group, TBAb changes were tracked in 6 patients, all of whom showed a loss of TBAb positivity. Persistent TBAb positivity was significantly more frequent in the Non-GD-hypo group than in the Non-GD-eu group (*P* = .033). Even in the Non-GD-hypo and Non-GD-eu groups, some patients exhibited persistent or newly developed TSAb positivity. In the Non-GD-hyper and Non-GD-swing groups, all patients were TSAb-positive at least once during the course of observation.

**Table 3:** Transition of TSHR-directed autoantibodies status and thyroid function outcomes in the non-GD group

	Transition of antibody status	Non-GD-hypo (*n* = 115)	Non-GD-eu (*n* = 17)	Non-GD-hyper (*n* = 2)	Non-GD-swing (*n* = 3)	*P*-value hypo vs eu	Follow-up period (years)
TBAb	(+) → (+)	17	0	0	2	.033	3.4 [0.4-11.6]
	(+) → (−)	18	6	1	1		
TSAb	(+) → (+)	7	1	2	2		3.3 [0.6-11.6]
	(+) → (−)	9	2	0	1		
	(−) → (+)	3	0	0	0		
	(−) → (−)	8	2	0	0		

Abbreviations: TBAb, thyrotropin receptor blocking antibody; TSAb, thyrotropin receptor–stimulating antibody.

Among the 41 patients in the Prior GD-hypo group whose TBAb levels were longitudinally evaluated, 27 (66%) remained positive and 14 (34%) became negative (Table 4). In the Prior GD-eu group, among the 7 patients with longitudinal data, 2 (29%) remained positive and 5 (71%) became negative. In the Prior GD-hyper group, all 4 patients with available data tested negative. The persistence of TBAb positivity was significantly more frequent in the Prior GD-hypo group than in the Prior GD-hyper group (*P* = .021). In the Prior GD-hypo and Prior GD-eu groups, some patients exhibited persistent or newly developed TSAb positivity.

**Table 4:** Transition of TSHR-directed autoantibodies status and thyroid function outcomes in the prior GD group

	Transition of antibody status	(I) Prior GD-hypo (*n* = 78)	(II) Prior GD-eu (*n* = 45)	(III) Prior GD-hyper (*n* = 16)	Prior GD-swing (*n* = 25)	*P*-value		*P*-value		Follow-up period (years)
						I vs II vs III	I vs II	I vs III	II vs III	
TBAb	(+) → (+)	27	2	0	2	.012	.10	.021	0.49	4.4 [0.5-14.3]
	(+) → (−)	14	5	4	5					
TSAb	(+) → (+)	17	4	4	7					3.7 [0.5-17.8]
	(+) → (−)	9	2	0	0					
	(−) → (+)	6	0	1	0					
	(−) → (−)	7	1	0	2					

Abbreviations: TBAb, thyrotropin receptor blocking antibody; TSAb, thyrotropin receptor–stimulating antibody

### Risk analysis for the hypothyroidism outcomes

In the Non-GD group, the risk factors for the hypothyroidism outcomes (Non-GD-hypo), compared with euthyroidism (Non-GD-eu), were examined using univariate logistic regression analysis. The TBAb status resulted in complete separation, as persistent positivity was observed exclusively in the Non-GD-hypo group, whereas TBAb became negative in all patients in the Non-GD-eu group (Table 3). Therefore, Firth's penalized likelihood method was applied. Using this approach, the persistence of TBAb positivity was associated with the hypothyroidism outcomes (OR, 3.51; 95% CI, 1.14-40.6; *P* = .013) (Table 5). Multivariate analysis was not performed because of the limited number of patients in the Non-GD-eu group and the complete separation. Given the heterogeneity in follow-up duration among patients, sensitivity analyses restricted to patients with an observation period of ≥3 years were performed, showing consistent results that confirm the robustness of the association between the persistence of TBAb positivity and the hypothyroidism outcomes (Table S2) [20].

**Table 5:** Risk factors for the hypothyroidism outcomes in the non-GD group

Variables	*n* (Non-GD-hypo vs Non-GD-eu)	Univariable analysis	*P*-value
Age (/10 years)	115 vs 17	0.93 [0.66-1.30]	.66
Sex (male to female)*^a^*	115 vs 17	2.86 [1.00-33.3]	.02
Follow-up period after TBAb measurement (years)	115 vs 17	0.92 [0.82-1.02]	.12
Transition of TBAb status ((+)→(+) to (+)→(−))*^a^*	35 vs 6	3.51 [1.14-40.6]	.013
FT4 (pmol/L)*^b^*	115 vs 17	0.48 [0.12-1.88]	.29
TSH (/10 µIU/mL)*^b^*	115 vs 17	1.05 [0.97-1.13]	.18
Thyroid autoantibodies			
TBAb (RIA) (/10%)	57 vs 11	1.14 [0.81-1.60]	.45
TBAb (EIA) (/10%)	52 vs 6	1.22 [0.84-1.78]	.32
TSAb (RIA) (/100%)*^b^*	57 vs 11	0.92 [0.33-2.59]	.88
TSAb (EIA) (/100%)*^b^*	52 vs 6	1.03 [0.61-1.75]	.91
TRAb (ECLIA) (/10 IU/L)*^b^*	89 vs 11	1.04 [0.69-1.56]	.86
TgAb (ECLIA) (/100 IU/mL)*^c^*	104 vs 15	1.03 [0.97-1.10]	.28
TPOAb (ECLIA) (/100 IU/mL)*^c^*	104 vs 15	1.10 [0.88-1.38]	.38
TV at the time of hypothyroidism diagnosis (/10 mL)	45 vs 3	1.12 [0.88-1.42]	.30

Abbreviations: TBAb, thyrotropin receptor blocking antibody; FT4, free thyroxine; TSH, thyrotropin; RIA, radioimmunoassay; EIA, enzyme immunoassay; TSAb, thyrotropin receptor–stimulating antibody; TRAb, thyrotropin receptor antibody; ECLIA, electrochemiluminescence immunoassay; TgAb, thyroglobulin antibody; TPOAb, thyroid peroxidase antibody; TV, thyroid volume

^*a*^Firth's penalized likelihood method was used for variables showing complete separation.

^*b*^Measured at the time of TBAb measurement.

^*c*^Measured at the first visit.

In the Prior GD group, the risk factors for the hypothyroidism outcomes (Prior GD-hypo) compared with nonhypothyroidism (Prior GD-non-hypo) were examined using logistic regression analysis. As in the Non-GD group, persistent TBAb positivity showed an association with the hypothyroidism outcomes, compared with nonhypothyroidism, in both univariate (OR, 6.75; 95% CI, 1.87-24.4; *P* = .0016) and multivariate analyses (OR, 6.88; 95% CI, 1.89-25.1; *P* = .0015) (Table 6). Sensitivity analyses restricted to patients with an observation period of ≥3 years were performed; consistent results that confirm the robustness of the association between the persistence of TBAb positivity and the hypothyroidism outcomes are provided in Table S3 [20]. In addition, high TgAb levels were significantly associated with the hypothyroidism outcomes (OR, 1.07; 95% CI, 1.02-1.13; *P* = .0004) (Table 6). Considering this result and the relationship between elevated TgAb and/or TPOAb levels and hypothyroidism due to Hashimoto's disease, TgAb and TPOAb may represent important confounders along with the follow-up period. Therefore, additional analyses were performed including transition of TBAb status and these autoantibody statuses as explanatory factors. As a result, persistent TBAb positivity remained significantly associated with hypothyroidism outcomes even after adjustment for elevated TgAb (Table S4) [20]. Furthermore, elevated TgAb was also independently associated with the hypothyroidism outcome. TPOAb was not associated with hypothyroidism outcomes, and positivity for either or both TgAb and TPOAb, which is included in the diagnostic criteria for Hashimoto's disease in Japan [30], was also not associated.

**Table 6:** Risk factors for the hypothyroidism outcomes in the prior GD group

Variables	*n* (Prior GD-hypo vs Prior GD-non-hypo)	Univariable analysis	*P*-value	Multivariable analysis	*P*-value
Age (/10 years)	78 vs 86	1.13 [0.91-1.41]	.27		
Sex (male to female)	78 vs 86	0.57 [0.24-1.38]	.21		
Follow-up period after TBAb measurement (years)	78 vs 86	0.98 [0.92-1.05]	.60	0.96 [0.84-1.09]	.55
Transition of TBAb status ((+)→(+) to (+)→(−))	41 vs 18	6.75 [1.87-24.4]	.0016	6.88 [1.89-25.1]	.0015
FT4 (pmol/L)*^a^*	78 vs 86	1.18 [0.49-2.85]	.71		
TSH (/10 µIU/mL)*^a^*	78 vs 86	1.01 [0.99-1.02]	.067		
Thyroid autoantibodies					
TBAb (RIA) (/10%)	38 vs 58	1.56 [1.21-2.02]	.0008		
TBAb (EIA) (/10%)	40 vs 26	1.00 [0.73-1.39]	.98		
TSAb (RIA) (/100%)*^a^*	38 vs 58	0.70 [0.48-1.01]	.046		
TSAb (EIA) (/100%)*^a^*	40 vs 26	0.88 [0.74-1.04]	.11		
TRAb (ECLIA) (/10 IU/L)*^a^*	61 vs 58	1.38 [1.08-1.75]	.0084		
TgAb (ECLIA) (/100 IU/mL)*^b^*	63 vs 73	1.07 [1.02-1.13]	.0004		
TPOAb (ECLIA) (/100 IU/mL)*^b^*	63 vs 73	1.12 [0.97-1.30]	.11		
TV upon hyperthyroidism (/10 mL)	15 vs 10	0.63 [0.34-1.17]	.059		

Abbreviations: TBAb, thyrotropin receptor blocking antibody; FT4, free thyroxine; TSH, thyrotropin; RIA, radioimmunoassay; EIA, enzyme immunoassay; TSAb, thyrotropin receptor–stimulating antibody; TRAb, thyrotropin receptor antibody; ECLIA, electrochemiluminescence immunoassay; TgAb, thyroglobulin antibody; TPOAb, thyroid peroxidase antibody; TV, thyroid volume

^*a*^Measured at the time of TBAb measurement.

^*b*^Measured at the first visit.

### Changes in TV after the diagnosis of hypothyroidism

**Figure 4:**
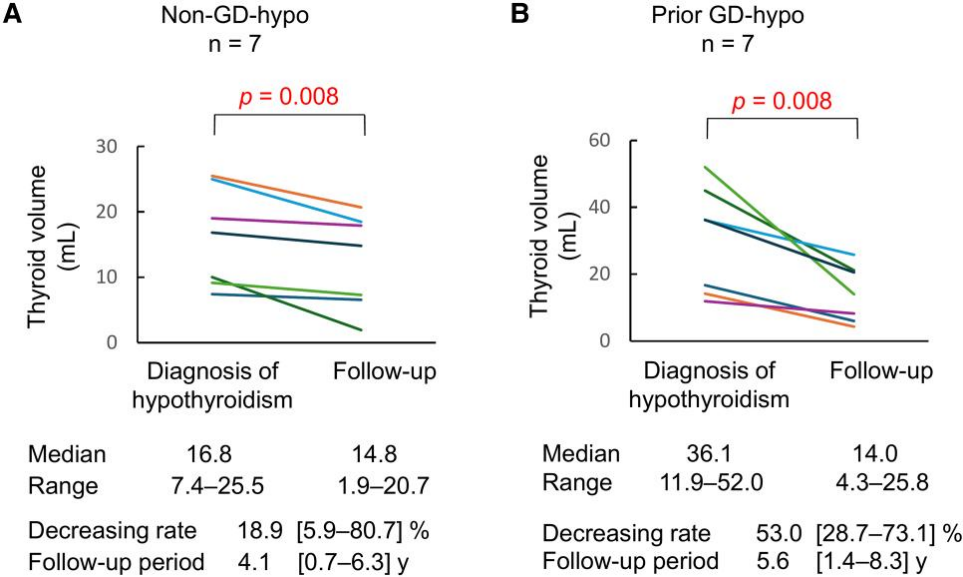
Changes in thyroid volume from the time of hypothyroidism diagnosis to follow-up in the Non-GD-hypo (A) and Prior GD-hypo (B) groups.

Data on changes in TV from the time of hypothyroidism diagnosis to follow-up were available for 7 patients each in the Non-GD-hypo and Prior GD-hypo groups. In the other groups, only a few patients had comparable data. In the Non-GD-hypo group, TV significantly decreased from 16.8 (7.4-25.5) mL to 14.8 (1.9-20.7) mL (*P* = .008), with a decreasing rate of 18.9% (5.9-80.7%) (Fig. 4A). In the Prior GD-hypo group, TV also significantly decreased from 36.1 (11.9-52.0) mL to 14.0 (4.3-25.8) mL (*P* = .008), with a decreasing rate of 53.0% (28.7-73.1%) (Fig. 4B).

## Discussion

This is the first large-scale investigation of the clinical outcomes of TBAb-positive patients with a long-term follow-up period (median 6.8 years). Most patients with TBAb-positive hypothyroidism remained hypothyroid for a long time, whereas those with prior GD who developed TBAb-positive hypothyroidism exhibited diverse thyroid functional outcomes. Persistent TBAb positivity was associated with the hypothyroidism outcomes in both groups.

In the Non-GD group, most patients (84%) remained hypothyroid, whereas 12% achieved euthyroidism. The development of hyperthyroidism was rare, occurring in only 3.7% of patients, including those classified into the Non-GD-hyper and Non-GD-swing groups. In a 1992 report by Takasu et al, among 21 patients with TBAb-positive hypothyroidism, hypothyroidism persisted in 15 (71%) patients, whereas 6 (29%) became euthyroid [10]. Similarly, in their 2012 report, among 34 patients with TBAb-positive hypothyroidism, 19 (56%) remained hypothyroid, 13 (38%) achieved euthyroidism, and 2 (6%) developed GD [18]. Although the proportion of patients with persistent hypothyroidism in this study was relatively higher, our findings are consistent with those of Takasu et al. These results suggest that although TBAb-positive hypothyroidism tends to persist over the long term, thyroid dysfunction can be reversed in a subset of patients. In the Prior GD group, thyroid functional outcomes were heterogeneous; however, the most frequent outcome was hypothyroidism (43%). When combined with euthyroidism (25%), 68% of the patients achieved remission. Relapse of hyperthyroidism was observed in only 22.8% of the patients, including those in the Prior GD-hyper and Prior GD-swing groups. These findings suggest that when patients with GD develop hypothyroidism in the presence of TBAb positivity, they are likely to achieve remission. However, careful follow-up is required because of the potential risk of relapse. Although block-and-replacement therapy is generally considered less favorable than the titration method owing to its higher incidence of adverse effects [31], it was chosen in 9.4% of the patients in this study. This suggests that, in the presence of TBAb, clinicians may prefer this regimen as a means of stabilizing fluctuating thyroid function.

In both the Non-GD and Prior GD groups, persistent TBAb positivity was associated with the hypothyroidism outcomes. Persistent TBAb positivity correlates with the hypothyroidism outcomes in patients with hypothyroidism without GD [10, 18], which is consistent with the findings of the present study. To the best of our knowledge, this is the first study to demonstrate that persistent TBAb positivity is also associated with the hypothyroidism outcomes in patients with GD. Persistent TBAb positivity may reflect a sustained predominance of TSH receptor–blocking activity over stimulatory components, thereby contributing to the persistence of hypothyroidism. However, the wide 95% CI indicates the limited precision of the effect estimate, likely owing to the small sample size. Therefore, the observed associations should be interpreted with caution and should be considered exploratory. Although variability in the observation period was considered an important potential confounder, the association between persistent TBAb positivity and the hypothyroidism outcomes remained robust in sensitivity analyses restricted to an observation period of ≥3 years and in multivariate analyses adjusting for the observation period in the Prior GD group. Because TgAb and TPOAb are related with hypothyroidism due to Hashimoto's disease, they may serve as important confounders, along with the observation period. In the Non-GD group, adjustment for confounding using multivariate analysis was not feasible due to the limited sample size. Although these autoantibodies tended to be higher in patients with hypothyroidism outcomes, no significant association was observed in the univariate analysis, suggesting that the impact of confounding is possibly small. In the Prior GD group, multivariate analysis showed that elevated TgAb level was independently associated with hypothyroidism outcomes, and persistent TBAb positivity remained associated with hypothyroidism outcomes even after adjustment for elevated TgAb levels, suggesting that chronic thyroiditis, in addition to TBAb, may potentially contribute to the development of hypothyroidism. In the Prior GD group, TBAb (RIA) was significantly higher than that in the Prior GD-hypo group, whereas no significant difference was observed in TBAb (EIA), suggesting limited clinical relevance in current practice. TV analysis was limited by the small number of patients for whom data were available.

Both the Non-GD-hypo and Prior GD-hypo groups exhibited significant longitudinal reductions in TV. However, owing to insufficient data, comparisons with other outcome groups could not be performed, and it remains unclear whether TV reduction is a risk factor for the hypothyroidism outcomes. In previous studies, patients with palpable goiters were more likely to experience the disappearance of TBAb and recovery from hypothyroidism, whereas those with an atrophic thyroid were more likely to show persistent TBAb positivity and sustained hypothyroidism [10, 18]. In addition, we previously demonstrated that among patients with GD who developed hypothyroidism, those positive for TBAb exhibited a greater reduction in TV than those without TBAb [29]. Taken together, these findings suggest that persistent TBAb positivity may contribute to the persistence of hypothyroidism through TV reduction. However, further studies with larger patient cohorts are required to validate this hypothesis.

TBAb functions as an antagonist of TSHR, and TSAb functions as an agonist. As these antibodies exert opposing effects, their coexistence within the same patient, together with temporal fluctuations in their respective titers, gives rise to complex clinical phenotypes. In the present study, all patients in the Non-GD-hyper and Non-GD-swing groups were positive for TSAb at the time of the diagnosis of hypothyroidism, indicating the coexistence of TBAb and TSAb (Table 1). One patient in the Non-GD-hyper group and 1 in the Non-GD-swing group became TBAb-negative during follow-up, whereas another patient in the Non-GD-swing group became TSAb-negative (Table 3). We speculate that fluctuations in the relative predominance of these antibodies may lead to corresponding changes in thyroid function. Although a subset of patients in the Non-GD-hypo and Non-GD-eu groups exhibited persistent or newly developed TSAb positivity, they did not develop hyperthyroidism, presumably because the effects of TBAb were more dominant than those of TSAb. Similarly, persistent or newly developed TSAb positivity was observed in the Prior GD-hypo and Prior GD-eu groups, with TBAb likely to be predominant over TSAb. Furthermore, Kahaly et al reported patients in whom TBAb appeared after the initiation of antithyroid drug therapy for GD and remission was subsequently achieved [32]. Taken together, when TBAb and TSAb coexist in the same patient, the balance between these antibodies and changes in their titers determine the outcomes of thyroid function and the occurrence of GD.

The present study suggests that measurement of TBAb levels during hypothyroidism provides useful prognostic information regarding future thyroid functional outcomes, regardless of the presence or absence of GD. A longitudinal assessment of the TBAb levels may further enhance its clinical utility. Although hypothyroidism caused by TBAb is often permanent, it can be reversible in some patients. Therefore, in patients with hypothyroidism of unknown etiology, information on TBAb positivity may aid in determining appropriate management strategies. Patients with GD who develop TBAb-positive hypothyroidism appear to have a higher likelihood of remission. When patients with GD develop hypothyroidism or exhibit fluctuations in thyroid function, the measurement of both TBAb and TSAb levels may be useful for elucidating the underlying pathophysiology and prognostic assessment. Furthermore, in women who wish to conceive or are pregnant and have hypothyroidism or autoimmune thyroid disease, TBAb measurement is warranted because TBAb can be transplacentally transferred to the fetus, posing a risk of fetal hypothyroidism [8, 12, 33, 34].

This study has certain limitations. First, selection bias may be present because this is a retrospective study conducted at a single specialized thyroid center in Japan. In addition, no predefined criteria were established for the measurement of TBAb or TV, with testing left to the discretion of the attending physicians. Second, the relatively small sample size may have limited the validity of the findings. Because the number of patients in whom longitudinal changes in TBAb levels could be assessed was limited, multivariate analysis could not be performed in the Non-GD group, and only 2 explanatory variables could be included in the Prior GD group. Consequently, adjustments for potential confounders may have been insufficient. In addition, the wide 95% CIs indicate limited precision of the effect estimates. However, given that opportunities to measure TBAb levels are generally limited in clinical practice, this cohort likely represents one of the largest single-center populations with TBAb measurements in Japan. Future multicenter or prospective studies are required to ensure adequately large sample sizes. Third, because there are multiple assay methods for measuring TBAb, TSAb, and TRAb levels depending on the measurement period, direct comparison of antibody titers was difficult, which may have affected the reliability of antibody positivity. Regarding the transition of TBAb and TSAb status, positive/negative classifications based on different assay methods were analyzed without distinction because of the limited sample size; therefore, the accuracy of these analyses may have been insufficient. Nevertheless, the concordance rate for positive/negative classification between TBAb (EIA) and TBAb (Bio) has been reported to be 91.5%, indicating a high level of agreement between the 2 assays [24]. Ideally, serum samples from all patients would have been stored frozen and TBAb would have been measured in all samples simultaneously using a single assay method. However, this was not feasible in practice. Therefore, in the present study, we retrospectively collected and analyzed TBAb data that had been obtained during outpatient care whenever measurement was considered clinically necessary. Fourth, because detailed information on the fundamental and clinical evaluation of the TBAb (RIA) and TBAb (EIA) assay kits was not available, the evidence supporting their analytical performance and the validity of the established cutoff values was limited. Finally, we were unable to perform adequately meaningful analyses of TV, which is considered an important factor for predicting thyroid functional outcomes, because data on TV at the time of hypothyroidism diagnosis and longitudinal changes in TV were available for only a limited number of patients.

In conclusion, this study clarified the clinical outcomes of thyroid function in patients with TBAb-positive hypothyroidism based on the presence or absence of GD. The outcome of this study indicates that persistent TBAb positivity was associated with the hypothyroidism outcomes.

## Data Availability

Some or all datasets generated during and/or analyzed during the current study are not publicly available but are available from the corresponding author on reasonable request.
